# Editorial: Immunosuppressive Amino Acid Catabolizing Enzymes in Heallth and Disease

**DOI:** 10.3389/fimmu.2021.689864

**Published:** 2021-05-05

**Authors:** Flavia Castellano, Jorge Correale, Valérie Molinier-Frenkel

**Affiliations:** ^1^ Univ Paris Est Creteil, INSERM, IMRB, Creteil, France; ^2^ AP-HP, Hopital Henri Mondor, Departement de Biologie-Pathologie, Creteil, France; ^3^ Department of Neurology, Fundacion para la Lucha Contra las Enfermedades Neurologicas de la Infancia (FLENI), Buenos Aires, Argentina

**Keywords:** immunosuppressive enzymes, amino acids (AA), immunoregulation, T cell response, B cell response, phenylalanine, tryptophan, arginin

Defective calibration of the immune response is involved in the pathophysiology of a great number of affections beyond chronic infections and autoimmunity. Membrane-expressed immune checkpoint molecules and cytokines are well-known mediators of immune regulation. Control of the level of some essential amino acids and production of bioactive amino acid-derived metabolites represents a less characterized pathway of immune regulation with potential for pharmacological manipulation (1).

Cells sense amino acid levels through at least two different pathways, involving the general control non-derepressible 2 (GCN2) kinase, and the mammalian target of rapamycin (mTOR) kinase, respectively (2). These two related serine/threonine kinases jointly assess nutritional deficiency (GCN2) and adequacy (mTOR). Increased amino acid catabolism leads to amino acid depletion, GCN2 activation and mTOR inactivation, thereby limiting activation of some immune cell populations, in particular T lymphocytes. Hence, basal amino acid catabolism can contribute to immune homeostasis, whereas elevated amino acid catalytic activity reinforces immune suppression. Furthermore, several downstream amino acid metabolites are also important biological mediators of immune response regulation.

Six major amino acid catabolizing enzymes endowed with immunosuppressive properties are known, which catabolize tryptophan [indole 2,3 dioxygenase (IDO) 1, IDO2, and tryptophan dehydrogenase (TDO) 2], arginine [arginase 1 and inducible nitric oxide synthase (iNOS)] and phenylalanine [Interleukin four-induced gene 1 (IL4I1)] (3). These enzymes are genetically unrelated, and their mechanisms of action are various and context-dependent. In the immune system, they are mainly produced by subpopulations of myeloid cells stimulated by specific signals, with certain species-related differences that complicate their study. This Research Topic gathers different contributions highlighting their activities in several pathological conditions, spanning from infection to autoimmunity, cancer, and atherosclerosis.

Three contributions address the role of tryptophan-degrading enzymes. The first article by Oliveira dos Santos et al. links tryptophan metabolism to cytokine production and parasitemia, in patients with malaria caused by Plasmodium vivax. In particular, the first malaria episode, which is characterized by a high parasite load, was associated with a stronger IFNγ response and higher levels of kynurenine (a product of the degradation of tryptophan by IDO or TDO) than subsequent episodes occurring after the development of specific IgG. This suggests induction of a regulatory circuit by IFNγ-induced IDO1 and/or TDO, which controls Th1 inflammation to avoid host immunopathology during the period preceding acquisition of protective humoral immunity.

In the second contribution, Merlo et al. address the complex issue of redundancy and complementarity of the two highly related enzymes, IDO1 and IDO2, by comparing T and B cell-mediated immune responses in double knock-out (KO) and single KOs mouse models in several contexts. Their data importantly show that single deletion of one of these genes leads to significant modifications of the expression of the other. This may affect interpretation of previous results of the literature, as IDO2, which has much weaker tryptophan degrading activity than IDO1, has been shown to play a proinflammatory role. Moreover, these models indicate that B cell responses directly depend on IDO2-induced inflammation, while IDO1 mediates T cell suppression in IDO2 KO mice, an effect that may be related to its enhanced expression in this model.

The last contribution on Trp catabolizing enzymes from Mohapatra et al. concentrates on the activity of TDO2 in the liver, which controls Trp plasmatic levels. They show that hepatocytes reroute Trp metabolism under hypoxic conditions by inhibiting TDO2 expression, which decreases kynurenine production, while tryptamine production by dopa decarboxylase is enhanced. However, AHR activation is maintained under hypoxia, as tryptamine is still capable to activate this important immunomodulatory pathway.

Two research papers reveal some unsuspected particularities of Arg1 and IL4I1. The work of Vonwirth et al. focuses on the activation and proliferation of T cells stimulated in supernatants of polymorphonuclear (PMN) cell cultures. Due to arginine depletion by arginase 1, T cell proliferation is abrogated in PMN supernatants. Surprisingly, specific inhibition of arginase 1 in PMN cultures not only releases T cell inhibition but leads to hyperactivation of the T lymphocytes, characterized by increased proliferation, cytotoxicity, and IL-9 and IL-17 production. The authors demonstrate that this hyperactivated state is induced by one or several low molecular weight (<3 kDA), high temperature-resistant PMN-derived small molecule(s), that are rapidly released after arginase 1 inhibition, although the exact nature of this (these) factor(s) is not identified.

The article from Puiffe et al. uses an IL4I1 KO mouse model to dissect the induction of the CD8 T cell response during an acute viral infection. Unexpectedly, the absence of the IL4I1 enzyme is associated with diminished expansion of functional short lived-effector CD8 T cells, but enhanced memory T cell differentiation. These observations are not related to intrinsic differences of CD8 T cells between WT and IL4I1 KO mice, but result from modulation of immune synapse formation and early activation events by IL4I1-expressing DCs. Indeed, IL4I1 enhances the T cell activation threshold, thereby favoring the priming of high-affinity clones, restriction of the response to the most immunodominant peptides, and rapid acquisition of effector differentiation.

An extensive review from Zaric et al. on the role of amino acids and amino-acid catabolizing enzymes points to their importance in the development of atherosclerosis. The review recapitulates available data indicating that IDO1 and arginase regulate inflammation in this context and should represent promising therapeutic targets in cardiovascular diseases.

Finally, although not included in this Research Topic, another nice review from Correale, appeared in Frontiers Immunology in January 2021, describes our knowledge on the role of these enzymes in autoimmunity through the example of multiple sclerosis, a disease that is considered to be mediated by autoreactive Th1, Th17, and B cells.

In conclusion, the six papers of this topic underline the complexity this family of enzymes and increase our knowledge in this complex field. We are grateful to all the authors and reviewers for their precious contributions to this Research Topic.

## Author Contributions

FC, VM-F, and JC wrote the editorial. All authors contributed to the article and approved the submitted version.

## Funding

This work was supported by a grant from the Fondation BMS pour la Recherche en Immuno-Oncologie (FC) and INCA N°2018-155 (VMF, FC).

## Conflict of Interest

The authors declare that the research was conducted in the absence of any commercial or financial relationships that could be construed as a potential conflict of interest.

## References

[1] Grohmann U Bronte V. Control of Immune Response by Amino Acid Metabolism: Metabolic Regulation of Immune Responses. Immunol Rev (2010) 236:243–64. 10.1111/j.1600-065X.2010.00915.x 20636821

[2] McGaha TL Huang L Lemos H Metz R Mautino M Prendergast GC. Amino Acid Catabolism: A Pivotal Regulator of Innate and Adaptive Immunity. Immunol Rev (2012) 249:135–57. 10.1111/j.1600-065X.2012.01149.x PMC4384693 22889220

[3] Molinier-Frenkel V Prévost-Blondel A Castellano F. The IL4I1 Enzyme: A New Player in the Immunosuppressive Tumor Microenvironment. Cells (2019) 8:757. 10.3390/cells8070757 PMC6678094 31330829

